# Accrual of organ damage in Behçet’s syndrome: trajectory, associated factors, and impact on patients’ quality of life over a 2-year prospective follow-up study

**DOI:** 10.1186/s13075-022-02947-y

**Published:** 2022-11-17

**Authors:** Alberto Floris, Matteo Piga, Riccardo Laconi, Gerard Espinosa, Giuseppe Lopalco, Luisa Serpa Pinto, Nikolaos Kougkas, Jurgen Sota, Andrea Lo Monaco, Marcello Govoni, Luca Cantarini, George Bertsias, João Correia, Florenzo Iannone, Ricard Cervera, Carlos Vasconcelos, Alessandro Mathieu, Alberto Cauli

**Affiliations:** 1 Department of Medical Sciences and Public Health, University of Cagliari, SS 554, 09042 Monserrato, CA Italy; 2 Rheumatology Unit, Azienda Ospedaliero-Universitaria of Cagliari, Monserrato, Italy; 3 Department of Autoimmune Diseases, University of Barcelona, Hospital Clinic, Institut d’Investigacions Biomediques August Pi i Sunyer (IDIBAPS), Barcelona, Catalonia Spain; 4 University of Bari, Rheumatology Unit, Bari, Italy; 5 Hospital Santo Antonio Centro Hospitalar do Porto, Unidade de Imunologia Clinica, Porto, Portugal; 6 Rheumatology, Clinical Immunology and Allergy Unit, University of Crete, Heraklion, Greece; 7 Fourth Department of Internal Medicine, School of Medicine, Hippokration Hospital, Thessaloniki, Greece; 8 Rheumatology Unit, University of Siena, Siena, Italy; 9 Rheumatology Unit - AOU, S. Anna, Ferrara, University of Ferrara, Ferrara, Italy; 10 University of Porto, UMIB Abel Salazar Biomedical Sciences Institute, Porto, Portugal

## Abstract

**Background:**

This study aimed to investigate the trajectory of damage accrual, associated factors, and impact on health-related quality of life (HR-QoL) in a multicenter cohort of patients with Behçet’s syndrome (BS) over 2 years of follow-up.

**Methods:**

Patients recruited in the BS Overall Damage Index (BODI) validation study were prospectively monitored for 2 years and assessed for damage accrual, defined as an increase ≥1 in the BODI score, and HR-QoL was evaluated by the SF-36 questionnaire. Logistic and multiple linear regression models were built to determine factors associated with damage accrual and impairment in the different SF-36 domains.

**Results:**

During follow-up, 36 out of 189 (19.0%) patients had an increase ≥1 in the BODI score with a mean (SD) difference of 1.7 (0.8) (*p* <0.001). The incidence rate of damage accrual was stable over time, regardless of the disease duration. Out of 61 new BODI items, 25 (41.0%) were considered related to glucocorticoid (GC) use. In multivariate analysis, duration of GC therapy (OR per 1-year 1.15, 95% CI 1.07–1.23; *p* <0.001) and occurrence of ≥1 disease relapse (OR 3.15, 95% CI 1.09–9.12; *p* 0.038) were identified as predictors of damage accrual, whereas the use of immunosuppressants showed a protective effect (OR 0.20, 95% CI 0.08–0.54, *p*<0.001). Damage accrual was independently associated with the impairment of different physical domains and, to a greater extent, in emotional domains of the SF-36 questionnaire. Female sex, higher disease activity, and fibromyalgia were also significantly associated with impairment in HR-QoL.

**Conclusion:**

In BS, organ damage accrues over time, also in long-standing disease, resulting in an impairment of the perceived physical and mental health. Adequate immunosuppressive treatment, preventing disease flares and minimizing exposure to GCs have a crucial role in lowering the risk of damage accrual.

**Supplementary Information:**

The online version contains supplementary material available at 10.1186/s13075-022-02947-y.

## Introduction

Behçet’s syndrome (BS) is a multisystem inflammatory disease characterized by a strong genetic background and a relapsing-remitting course [1, 2]. In patients affected with BS, both disease activity and treatment may lead to the accrual of organ damage, defined as any irreversible anatomic or functional alteration potentially resulting in impaired quality of life and increased morbidity and mortality [2, 3].

According to the European Alliance of Associations for Rheumatology (EULAR) recommendations, preventing the accrual of organ damage is a primary goal in treating BS patients [4]. However, although this is one of the most widely accepted and shared general principles, there is still insufficient knowledge about the incidence, trajectory, qualitative features, and risk factors of damage accrual in BS. Moreover, no direct data on how damage affects other short- and long-term outcomes, such as quality of life (QoL), are currently available.

In this context, the recent development of Behçet’s syndrome Overall Damage Index (BODI), the first damage assessment tool specifically designed for BS, may favour significant advances in the knowledge about the characteristics and outcomes of damage accrual, supporting the design of effective preventive strategies [3].

In a preliminary cross-sectional study, male sex, longer disease duration, major organ involvement, and lack of use of TNF inhibitors were significantly associated with the presence of damage evaluated using the BODI [3]. Conversely, no significant association was recorded between the extent of damage and the impairment of health-related quality of life (HR-QoL) [3]. However, the cross-sectional nature of this study prevented assessing the temporal link between damage accrual and impairment of HR-QoL. Indeed, a longitudinal study design is needed to evaluate the incidence of organ damage accrual, predictive factors, and associated short- and long-term outcomes [5].

The present study reports on the 2-year follow-up extension of the BODI validation cohort, aiming to investigate the trajectory of damage accrual, associated factors, and impact on HR-QoL in patients with BS.

## Methods

### Study design and outcomes

The present study included and followed up for 2 years the patients enrolled in the BODI validation study, a multicenter cohort of BS patients recruited according to the following inclusion criteria: (a) diagnosis of BS fulfilling the International Study Group (ISG) criteria [6] or the International Criteria for Behçet’s Disease (ICBD) [7], (b) disease duration ≥12 months, (c) age at enrollment ≥18 years and (d) ability to provide informed consent.

Active and cumulative clinical manifestations, previous and ongoing medications, and overall disease activity assessed by the Behçet’s Disease Current Activity Form (BDCAF) [8], the Physician Global Assessment (PGA) [9], and the Patient Global Assessment (PtGA) [9] were recorded during follow-up. Furthermore, disease relapses, defined by any treatment escalation due to disease activity during follow-up, were recorded. The HR-QoL and the extent and type of organ damage were recorded at baseline and 2-year follow-up visits.

The HR-QoL was assessed by the SF-36 questionnaire, which consists of a multipurpose, generic (no disease-specific) short-form health survey with only 36 questions. It yields an eight-scale profile of scores as well as physical and mental health summary measures (higher scores correspond to higher HR-QoL) [10]. In this study, all eight domains of the SF-36 questionnaire were assessed: physical functioning (PF), role physical (RP), bodily pain (BP), general health (GH), mental health (MH), vitality (VT), social functioning (SF), and role emotional (RE). The results were then summarized in the Physical Component Summary (PCS), measuring the physical domains of QoL (PF, RP, BP, GH), and in the Mental Component Summary (MCS), measuring the emotional domains of QoL (SF, VT, RE, MH) [10].

The extent and type of damage were assessed by the BODI, consisting of 34 items and 12 subitems, categorized into 9 organ/system domains: mucocutaneous, musculoskeletal, ocular, vascular, cardiovascular, neuropsychiatric, gastrointestinal, reproductive system and miscellaneous. Each item and subitem score 1 point, with the total score ranging from 0-46. In the present study, damage accrual was defined as a ∆-BODI ≥1, calculated by subtracting the baseline BODI score from the score recorded at the 2-year follow-up visit. Furthermore, to evaluate the effect of glucocorticoids (GCs) on damage accrual, the individual BODI items were a priori classified into the following categories: definitely related to glucocorticoid (GC) therapy (i.e. diabetes, cataract, osteoporotic fractures or vertebral collapse, avascular necrosis, diabetes), possibly related to GCs (i.e. muscle atrophy, ischaemic heart disease), and independent of GCs (e.g. pulmonary aneurysms, myelopathy), taking into account underlying adverse effects [11, 12].

### Statistical analysis

Categorical variables were expressed as absolute values and frequencies (%). Normally and nonnormally distributed continuous variables are reported as the mean ± standard deviation (SD) and median and interquartile range (IQR), respectively.

The chi-squared test or Mann–Whitney *U*-test was used in univariate analysis to identify potential risk factors for damage accrual. Then, a multivariate model for stepwise logistic regression included factors with a *p*-value < 0.1 in univariate analysis. Odds ratios (ORs) with 95% confidence intervals (95% CIs) were calculated. Multiple linear regression models were built to assess the relationship between organ damage accrual and HR-QoL, including the individual and composite SF-36 domains as dependent variables and ∆-BODI ≥1 as independent variables. The models included age, sex, disease activity, fibromyalgia, glucocorticoid duration, and major organ involvement as potential confounding factors. The results were reported as a beta (B) coefficient. Statistical significance was set at *p*-value < 0.05.

## Results

Based on the availability and completeness of the follow-up data (Supplementary Fig. 1), out of the 228 patients recruited in the original BODI validation cohort, 189 were enrolled for the analysis of the incidence of damage accrual and associated factors (Table 1), and 147 were enrolled for the assessment of the relationship between damage accrual and HR-QoL (Supplementary Table).

**Table 1:** Baseline features of the extension BODI cohort (*n*=189)

**Demographics**	
Male gender, *n* (%)	92 (48.7)
Age at enrolment, mean (SD) years	46.2 (12.1)
Age at the disease onset, mean (SD) years	32.6 (11.3)
Age at diagnosis, mean (SD) years	35.5 (11.1)
Disease duration, mean (SD) years	10.8 (8.3)
**Cumulative clinical manifestations**	
Oral aphtosis, *n* (%)	186 (98.4)
Genital aphtosis, *n* (%)	137 (72.5)
Skin lesions, *n* (%)	137 (72.5)
Ocular manifestations, *n* (%)	108 (57.1)
Neurologic lesions, *n* (%)	37 (21.8)
Vascular lesions, *n* (%)	41 (21.7)
Pathergy test, *n* (%)	25 (13.2)
Arthritis, *n* (%)	107 (57.5)
Gastrointestinal manifestation, *n* (%)	27 (14.7)
**Active clinical manifestations**	
Oral aphtosis, *n* (%)	63 (33.3)
Genital aphtosis, *n* (%)	12 (6.3)
Skin lesions, *n* (%)	20 (10.6)
Ocular manifestations, *n* (%)	12 (6.4)
Neurologic lesions, *n* (%)	5 (2.7)
Vascular lesions, *n* (%)	3 (1.6)
Arthritis, *n* (%)	17 (9.3)
Gastrointestinal manifestation, *n* (%)	9 (5.0)
**Ongoing treatment**	
Glucocorticoid ongoing, *n* (%)	93 (49.2)
Glucocorticoid duration, mean (SD) months	54.9 (66.6)
Conventional Immunosuppressants^a^, *n* (%)	81 (42.9)
TNF inhibitors, *n* (%)	44 (23.3)
**Disease activity**	
BDCAF, mean (SD) score	2.7 (2.8)
PGA, mean (SD) cm	2.0 (2.1)
PtGA, mean (SD) cm	2.8 (2.6)
**Damage**	
BODI, mean (SD) score	1.6 (2.0)
BODI ≥1, *n* (%)	114 (60.3)

*BODI* Behçet’s syndrome Overall Damage Index, *BDCAF* Behçet’s Disease Current Activity Form, *PGA* physician’s global assessment of disease activity, *PtGA* patient’s global assessment of disease activity

^a^Conventional immunosuppressants: azathioprine, methotrexate, cyclophosphamide, cyclosporine A, sulfasalazine, thalidomide

**Fig. 1:**
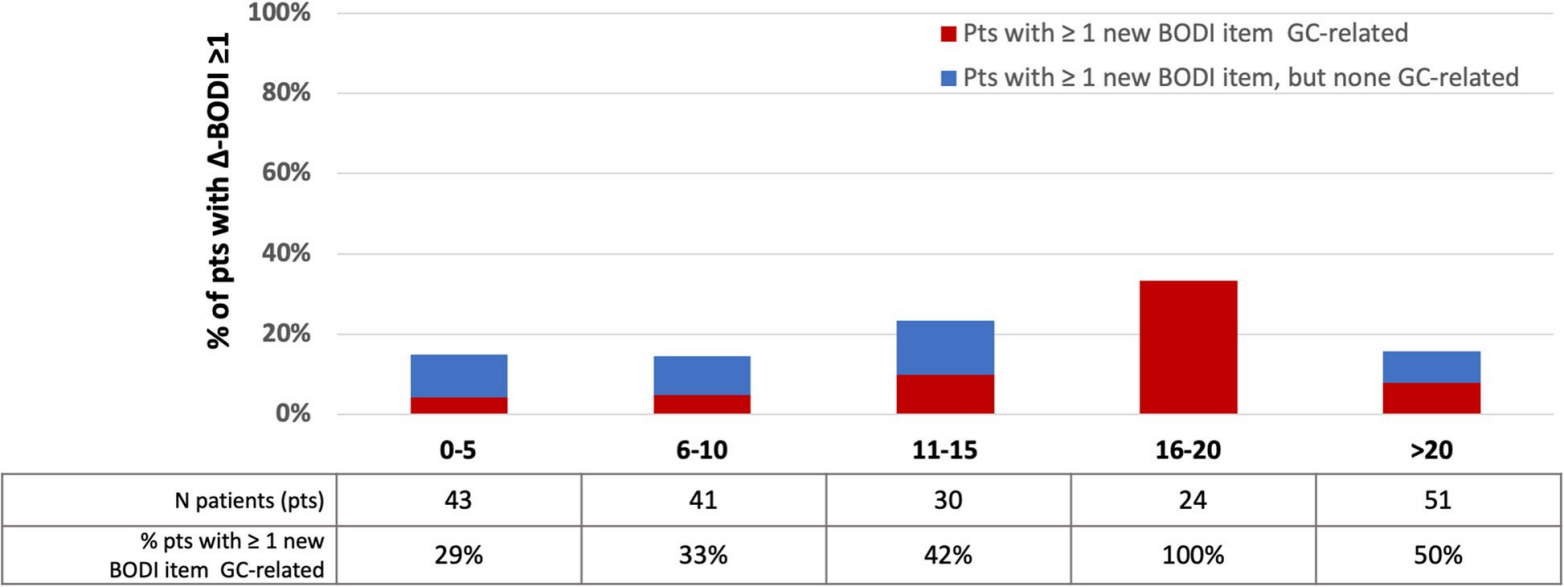
Rate of patients experiencing accrual of organ damage over the 2 years of follow-up according to the disease duration at enrolment. Pts, patients. BODI, Behçet’s syndrome Overall Damage Index; GC, glucocorticoids

**Fig. 2:**
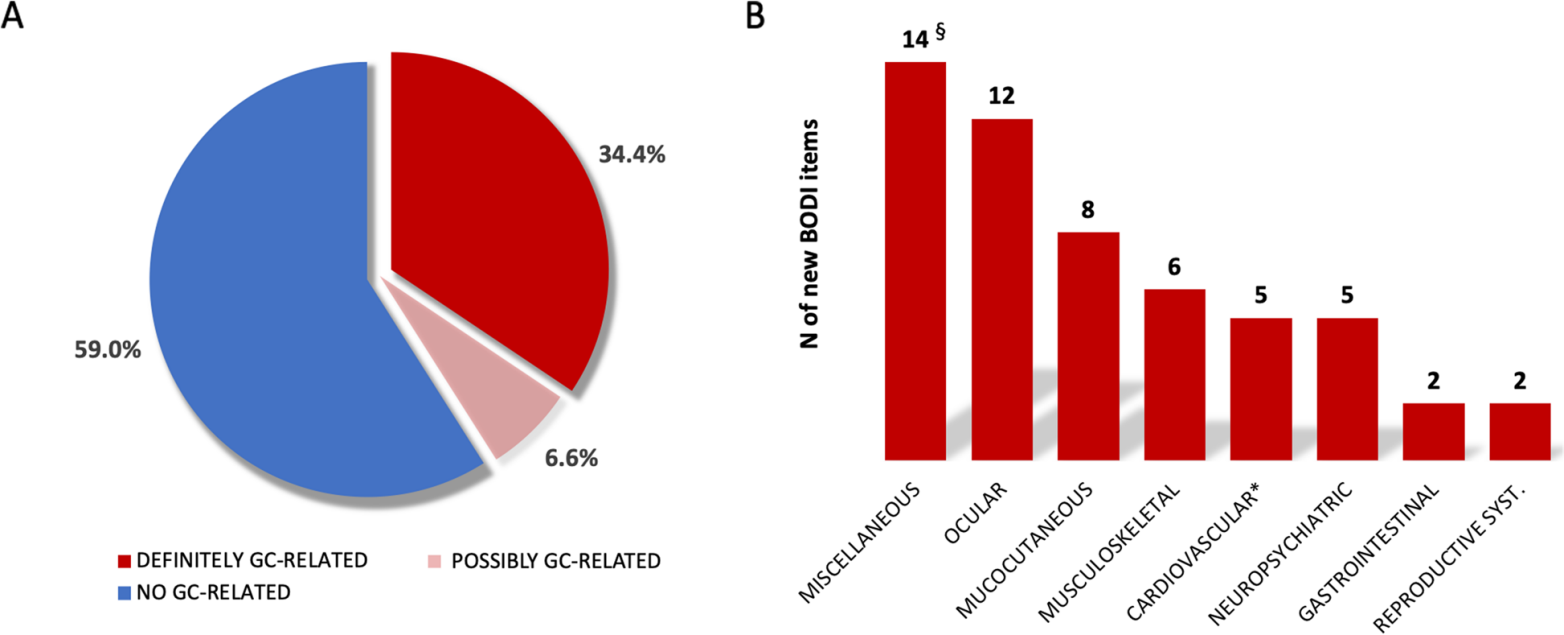
Type of damage accrual classified according to **A** the potential relationship with glucocorticoid treatment and **B** the involved organs/systems (BODI domains). GC, glucocorticoid; BODI, Behçet’s Syndrome Overall Damage index. § 13/14 Diabetes. *Cardiac and vascular BODI domains were merged

During the 2-year follow-up, the mean (SD) BODI score increased from 1.6 (2.1) to 1.9 (2.1), with a mean (SD) ∆-BODI of 0.3 (0.8) points (*p* <0.001). Thirty-six (19.0%) patients had an increase ≥1 in the BODI score, with a mean ∆-BODI of 1.7 (0.8) (*p* <0.001). In addition, the number of patients with at least one item of damage increased from 114 (60.3%) to 131 (69.3%). When the patients were stratified according to disease duration, the incidence of organ damage accrual remained stable, with no significant differences between the different groups (*p* 0.255), although a trend of a relative increase in the percentage of patients with at least one GC-related item of damage was recorded (Fig. 1). Overall, 61 new BODI items were recorded, with diabetes (21.3%), mucocutaneous scars (11.5%), visual impairment (11.5%), and osteoporotic fracture or vertebral collapse (8.2%) being the most frequent (Table 2). Twenty-five (41.0%) new BODI items were classified as definitely or possibly related to GC therapy (Fig. 2A). Figure 2B represents the distribution of the new damage items into the different BODI system/organ domains.

**Table 2:** Prevalence of the new BODI items occurred over the 2 years of FU

New BODI items	*N*=61
Diabetes^a^	13 (21.3%)
Mucocutaneous scars	7 (11.5%)
Visual impairment in one eye	7 (11.5%)
Osteoporotic fracture or vertebral collapse^a^	5 (8.2%)
Motor or sensory disturbance	4 (6.6)
Cataract^a^	3 (4.9)
Skin ulceration	2 (3.3)
Muscle atrophy^a^	2 (3.3)
DVT	2 (3.3)
DVT occurred > 1 episode	2 (3.3)
Ischaemic heart disease^a^	2 (3.3)
Peripheral neuropathy	2 (3.3)
Stricture	2 (3.3)
Premature gonadal failure	2 (3.3)
Anterior segment change	1 (1.6)
Posterior segment change	1 (1.6)
Second eye	1 (1.6)
Psychiatric disturbance	1 (1.6)
Infarction or resection of any part of gastrointestinal tract	1 (1.6)
Malignancy	1 (1.6)

*BODI* Behçet’s syndrome Overall Index, *DVT* deep venous thrombosis

^a^ Items of damage definitely or possibly related to glucocorticoid therapy

### Risk factors of damage accrual over 2 years follow-up

According to the univariate analysis, factors associated with the accrual of organ damage were older age at enrolment (median 56.2, IQR 42.9–62.0 vs median 46.6, IQR 35.4–53.1 years; *p* = 0.001), more prolonged exposure to GC therapy (median 9.3 years, IQR 2.2–12.3 vs median 2.0 years, IQR 0.7–6.0; *p* <0.001), and occurrence of at least one relapse (25.0% vs 13.1%; *p* = 0.07). Conversely, patients on treatment with conventional synthetic and biologic immunosuppressants showed a lower likelihood of damage accrual (66.7% vs 86.9%; *p* 0.004) (Table 3).

**Table 3:** Uni- and multivariate analysis exploring factors associated with increased risk of damage accrual

	Univariate analysis			Multivariate analysis	
	∆-BODI ≥1 (***n*** 36)	∆-BODI = 0 (***n*** 153)	***p***	OR (95%CI)	***p***
Male gender	16 (44.4%)	76 (49.7%)	0.572		
Age at enrolment	56.2 (42.9–62.0)	46.6 (35.4–53.1)	**0.001**	---	---
Disease duration	12.9 (7.1–17.5)	11.1 (5.4–21.2)	0.483		
Major organ involvement^a^	22 (61.1%)	72 (47.1%)	0,129		
BDCAF at baseline	3 (0–5)	2 (0–5)	0.365		
BDCAF at follow-up visit	3 (3–5)	3 (0–7)	0.188		
Glucocorticoid duration	9.3 (2.2–12.3)	2.0 (0.7–6.0)	**<0.001**	1.150 (1.072–1.234)	**<0.001**
Immunosuppressant^b^	24 (66.7%)	133 (86.9%)	**0.004**	0.201 (0.076–0.536)	**<0.001**
Cumulative n of immunosuppressants	2 (1–2)	2 (1–3)	0.162		
At least 1 relapse	9 (25.0%)	20 (13.1%)	**0.070**	3.146 (1.085–9.120)	**0.038**
BODI score at baseline	1.0 (0–2.0)	1 (0–2)	0.579		

In the univariate analysis, continuous variables are expressed as median (IQR) and categorical variables as number (%). Interval—age, disease duration and glucocorticoids refer to years. *BODI*, Behçet’s syndrome Overall Damage Index; *BDCAF*, Behçet’s Disease Current Activity Form; *OR*, odds ratio

^a^ Cumulative vascular, neurologic, gastrointestinal manifestations

^b^ Conventional and biologic immunosuppressants: azathioprine, methotrexate, cyclophosphamide, cyclosporine A, sulfasalazine, thalidomide, TNF-alpha inhibitors

In the multivariate analysis, the duration of GC therapy (OR per 1-year 1.15, 95% CI 1.07–1.23; *p* <0.001) and the occurrence of at least one relapse (OR 3.15, 95% CI 1.09–9.12; *p* = 0.038) were confirmed as independent risk factors for damage accrual, whereas the use of conventional or biologic immunosuppressants had an independent protective effect (OR 0.20, 95% CI 0.08–0.54, *p*<0.001) (Table 3).

### Relationship between damage accrual and HR-QoL

**Fig. 3:**
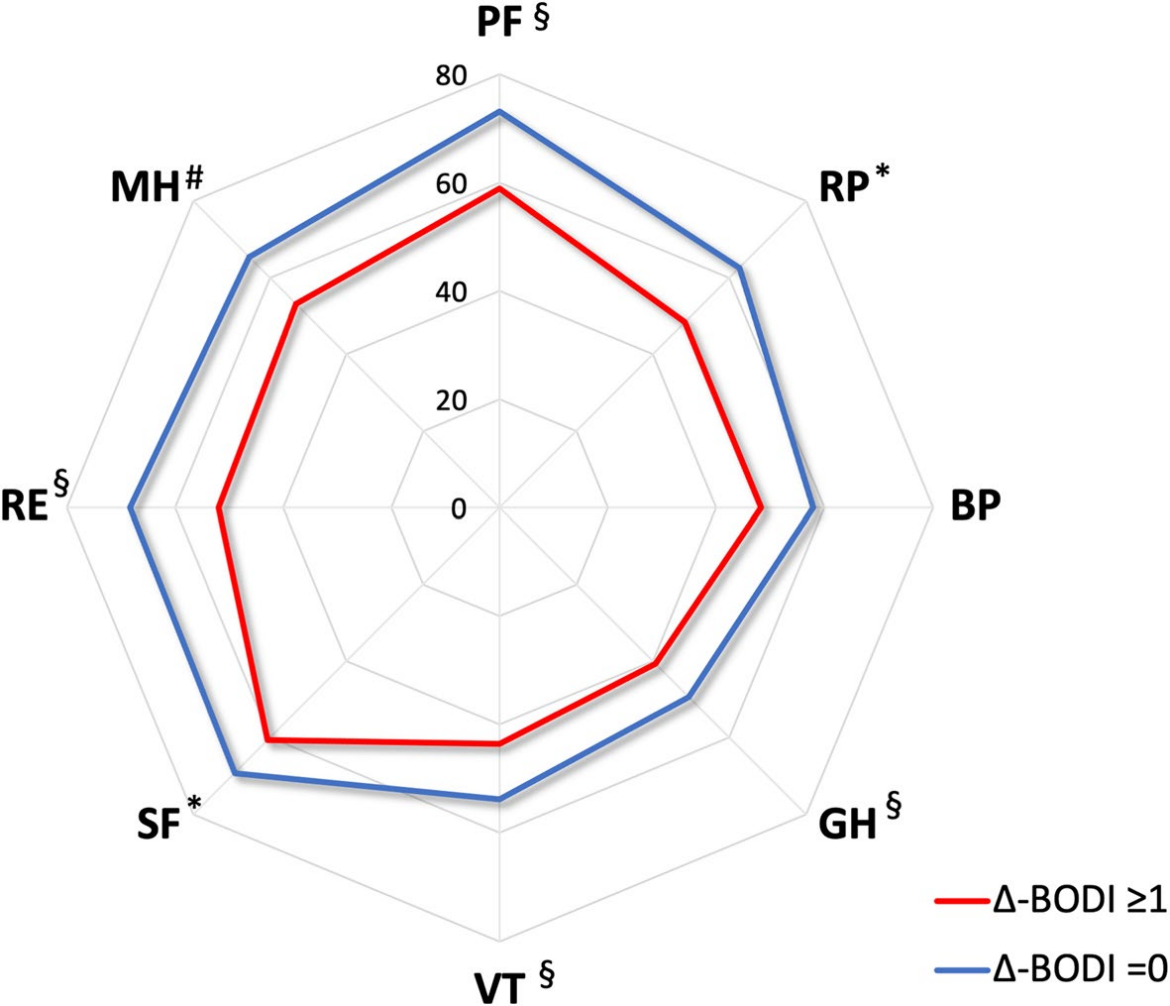
Radar plot representing the mean values of each SF-36 domain in patients having (∆-BODI ≥1) or not (∆-BODI=0) accrual of organ damage over the 2 years of follow-up. PF, physical function; RP, role-physical; BP, body pain; GH, general health; VT, vitality; SP, social function; RE, role emotional; MH, mental health. * *p* <0.05, § *p* <0.01, # *p* <0.001 in multiple regression

In the multiple regression analysis, a significant correlation was recorded between damage accrual and impairment in the PF (B-coefficient [B] = − 13.21, *p*-value 0.005) and GH (B − 11.91, *p* = 0.004) physical domains of the SF-36 questionnaire. However, the most relevant associations were found between damage accrual and impairment of the RP (B − 12.39, *p* = 0.018), VT (B − 12.13, *p* = 0.006), SF (B − 10.59, *p* = 0.035), RE (B − 16.51, *p* = 0.001), and MH (B − 14.87, *p* <0.001) emotional domains of the SF-36 questionnaire, including the MCS (B − 6.90, *p* = 0.021) (Table 4, Fig. 3). Female sex, higher disease activity by BDCAF, and fibromyalgia were also independently associated with HR-QoL impairment evaluated by the SF-36 questionnaire (Table 4).

**Table 4:** Results of multiple linear regression (B coefficents) exploring factors independently associated with HR-QoL

	SF-36 Domains									
	PF	RP	BP	GH	VT	SF	RE	MH	PCS	MCS
**∆-BODI ≥1**	− 13.21^§^	− 12.39^†^	--	− 11.91^§^	− 12.13^§^	− 10.59^†^	− 16.51^§^	− 14.87^#^	--	− 6.90^†^
**Female**	− 10.76^§^	− 15.86^#^	− 19.58^#^	− 10.55^§^	− 14.77^#^	− 17.47^#^	− 13.33^§^	--	− 6.42^#^	− 4.24^§^
**Fibromyalgia**	− 30.07^#^	− 33.27^#^	− 26.19^§^	− 24.24^#^	− 22.29^§^	− 27.48^§^	− 20.56^†^	− 25.31^§^	− 10.56^#^	− 9.49^†^
**BDCAF**	− 1.25^†^	− 2.11^§^	− 2.493^#^	− 2.89^#^	− 2.91^#^	− 1.60^†^	− 1.83^§^	− 1.91^#^	− 0.77^§^	− 0.95^#^
**Age**	− 0.51^#^	--	− 0.58^#^	--	--	--	--	--	− 0.16^#^	--
**GC duration**	--	− 0.140^#^	--	--	--	--	− 0.09^†^	--	− 0.03^†^	--
**Major organ involvement**	--	--	--	--	--	--	--	--	--	--
**Immunosuppressants**^a^	--	--	--	--	--	--	--	--	--	--

*HR-QoL* health-related quality of life, *SF-36* Short form 36 questionnaire for assessment of HR-QoL, *PF* physical functioning, *RP* role physical, *BP* bodily pain, *GH* general health, *MH* mental health, *VT* vitality, *SF* social functioning, *RE* role emotional, *MCS* physical component summary (PCS), *MCS* mental component summary, *GC* glucocorticoids

^a^ Conventional and biologic immunosuppressants: azathioprine, methotrexate, cyclophosphamide, cyclosporine A, sulfasalazine, thalidomide, TNF-alpha inhibitors

† *p* <0.05, ^§^ *p* <0.01, ^#^ *p* <0.001

## Discussion

This study provides original and meaningful data regarding the incidence, type of damage accrual, and associated factors in a multicenter cohort of BS patients prospectively followed up for 2 years. Moreover, the present study demonstrated how organ damage accrual is significantly associated with a decline in HR-QoL, primarily emotional but, to a lesser extent, also physical.

In the BODI cohort, the incidence of damage accrual was remarkable and stable over time, ranging approximately 20% regardless of the disease duration. The stable trend of damage accrual, even in patients with long-standing disease, may seem to be in contrast with the most common type of BS course, in which disease activity and severity tend to abate over time [13]. A partial explanation of this phenomenon may be the prolonged exposure to GC treatment, which was found to be a factor associated with damage accrual independent of other variables, such as disease duration and major organ involvement or the number of immunosuppressants (surrogates of disease severity and resistance to treatment, respectively). Furthermore, when the type of damage was analysed, it was found that approximately 40% of the newly recorded BODI items were definitely or possibly related to GC treatment, and the percentage of patients with at least one item of GC-related damage increased over time.

Consistent with the described data and the provided interpretation, a longer exposure to GC and an inadequate disease activity control needing an escalation of treatment were identified as independent risk factors for damage accrual. In contrast, the use of immunosuppressants showed a protective effect. The lack of an association between major organ involvement and damage accrual in this study cohort may also be due to the long-standing nature of our BS cohort and the consequent low prevalence of ongoing active visceral involvement. Indeed, the incidence of damage due to disease activity may be significantly higher in the earlier stage of the BS course [14, 15]. Noteworthy, the observed lack of association between damage accrual and the BDCAF, measured at the baseline and follow-up visit, does not mean that disease activity is not a determinant of damage. This is because the damage is also strongly related to the duration of disease activity. Thus, the assessment of disease activity at single time points may prevent capturing its association with damage accrual.

Finally, this study provides the first evidence regarding the influence of damage accrual on the impairment of HR-QoL, which is included in the outcomes core set for the monitoring and management of BS [16]. In the cross-sectional phase of the BODI validation study, no correlation between the extent of damage expressed as the total BODI score and the concomitant mental and physical domains of the SF-36 was found [3]. However, we speculated that this lack of association might be due to coping mechanisms, as observed in other chronic diseases [17, 18]. Following this assumption, in the present longitudinal extension of the BODI project, we found that the accrual of organ damage, rather than its extent assessed in a single visit, was associated with impairment of different aspects of HR-QoL, especially those mental related.

This study has several strengths. It first addressed the topic of damage accrual in BS as a prospective analysis and simultaneously analysed its determinant and outcomes, assuring higher completeness and consistency of the provided model. In addition, the risk factors and damage outcomes analysis was adjusted for major confounding covariates and provided coherent results with a fully rational explanation. Finally, this study provides data in support of the criterion validity of the BODI. Indeed, although a standardized and validated definition of a minimally important change in the BODI score is not currently available, this study showed that an increase of ≥1 has a relevant impact on patient quality of life.

Some limitations are also present in this study. The long-standing nature of the studied cohort and the relatively low proportion of patients with “major organ involvement” (which may be considered a surrogate of disease severity) may prevent a complete and time-related description of the trajectory of organ damage accrual. In addition, the definition of relapses as an increase in treatment due to disease activity is not validated in BS, although it may be considered a valid surrogate. Finally, further validation of the BODI in wider and ethnically heterogeneous cohorts, unrelated to the original BODI cohort, with earlier disease and longer follow-up duration is needed.

In conclusion, this study highlights how measuring and targeting organ damage is crucial in the management of BS to prevent its progressive accrual and the related impairment of perceived physical and mental health. This is especially relevant considering the need to design strategies for patient empowerment and self-management [19]. From this perspective, the use of an adequate immunosuppressive therapy, minimizing steroid exposure, and lowering the risk of relapse seem to have a key role in preventing damage accrual. However, further research in larger inception cohorts of BS patients followed for a longer time is needed to validate these results.

## Supplementary Information

**Additional file 1: Supplementary Figure 1.** Flow chart representing the patients’ recruiting process from the BODI validation cohort.

**Additional file 2: Supplementary Table 1.** Baseline features of the extension BODI cohort recruited for the analysis of the association between damage accrual and Heath related quality of life (*n*=147).

## Data Availability

The datasets analysed during the current study are available from the corresponding author upon reasonable request.
