# Supplementary figures and images for: Accrual of organ damage in Behçet’s syndrome: trajectory, associated factors, and impact on patients’ quality of life over a 2-year prospective follow-up study
Source: Arthritis Res Ther. 2022 Nov 17;24:253. doi: 10.1186/s13075-022-02947-y (PMC9670626; doi:10.1186/s13075-022-02947-y)

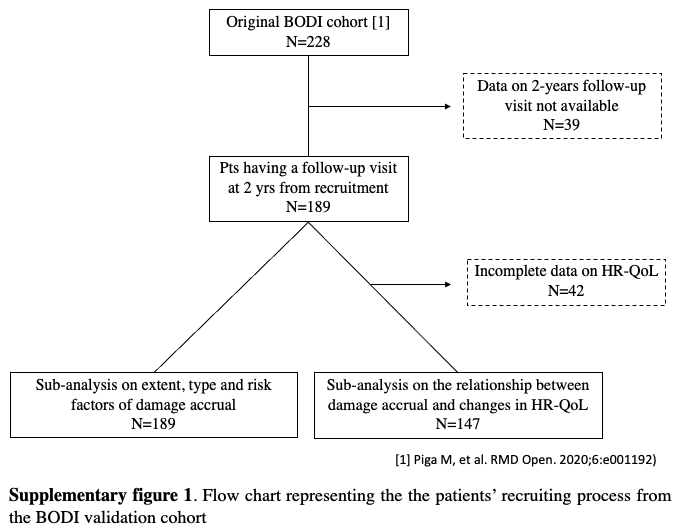

Supplement: Supplementary file 1 — Additional file 1: Supplementary Figure 1. Flow chart representing the patients’ recruiting process from the BODI validation cohort. [file 13075_2022_2947_MOESM1_ESM.docx]
