# Supplementary material for: Accrual of organ damage in Behçet’s syndrome: trajectory, associated factors, and impact on patients’ quality of life over a 2-year prospective follow-up study
Source: Arthritis Res Ther. 2022 Nov 17;24:253. doi: 10.1186/s13075-022-02947-y (PMC9670626; doi:10.1186/s13075-022-02947-y)
Supplement: Supplementary file 2 — Additional file 2: Supplementary Table 1. Baseline features of the extension BODI cohort recruited for the analysis of the association between damage accrual and Heath related quality of life (n=147). [file 13075_2022_2947_MOESM2_ESM.docx]

| **Supplementary Table 1.** Baseline features of the extension BODI cohort recruited for the analysis of the association between damage accrual and Heath related quality of life (n=147). | |
| --- | --- |
| **Demographics** |  |
| Male gender, n (%) | 74 (50.3%) |
| Age at enrolment, mean (SD) yrs | 41.2 (12.4) |
| Age at the disease onset, mean (SD) yrs | 32.7 (11.8) |
| Age at diagnosis, mean (SD) yrs | 35.6 (11.4) |
| Disease duration, mean (SD) yrs | 13.43 (10.1) |
| **Cumulative clinical manifestations** |  |
| Oral aphtosis, n (%) | 146 (99.3%) |
| Genital aphtosis, n (%) | 109 (74.1%) |
| Skin lesions, n (%) | 114 (77.6%) |
| Ocular manifestations, n (%) | 86 (58.5%) |
| Neurologic lesions, n (%) | 28 (19.2%) |
| Vascular lesions, n (%) | 30 (20.4%) |
| Pathergy test, n (%) | 22 (15.0%) |
| Arthritis, n (%) | 94 (63.9%) |
| Gastrointestinal manifestation, n (%) | 24 (16.8%) |
| **Active clinical manifestations** |  |
| Oral aphtosis, n (%) | 53 (37.3%) |
| Genital aphtosis, n (%) | 8 (5.8%) |
| Skin lesions, n (%) | 14 (10.1%) |
| Ocular manifestations, n (%) | 4 (2.9%) |
| Neurologic lesions, n (%) | 2 (1.4%) |
| Vascular lesions, n (%) | 0 (0%) |
| Arthritis, n (%) | 21 (14.9%) |
| Gastrointestinal manifestation, n (%) | 7 (5.1%) |
| **Ongoing treatment** |  |
| Glucocorticoid ongoing, n (%) | 51 (34.7%) |
| Glucocorticoid duration, mean (SD) months | 55 (62) |
| Conventional Immunosuppressants, n (%) | 60 (40.8%) |
| TNF inhibitors, n (%) | 40 (27.2%) |
| **Disease activity** |  |
| BDCAF, mean (SD) score | 3.1 (3.2) |
| PGA, mean (SD) score | 2.3 (2.1) |
| PtGA, mean (SD) score | 3.0 (2.4) |
| **Damage** |  |
| BODI, mean (SD) score | 1.9 (2.0) |
| BODI ≥1, n (%) | 88 (59.9%) |
| BODI, Behçet's syndrome Overall Damage Index. BDCAF, Behçet’s Disease Current Activity Form. PGA, physician’s global assessment of disease activity. PtGA, patient’s global assessment of disease activity | |
